# Effects of Statin versus the Combination of Ezetimibe plus Statin on Serum Lipid Absorption Markers in Patients with Acute Coronary Syndrome

**DOI:** 10.1155/2015/109158

**Published:** 2015-03-01

**Authors:** Erisa Watanabe, Junichi Yamaguchi, Hiroyuki Arashi, Hiroshi Ogawa, Nobuhisa Hagiwara

**Affiliations:** Department of Cardiology, The Heart Institute of Japan, Tokyo Women's Medical University, 8-1 Kawada-cho, Shinjuku-ku, Tokyo 1628666, Japan

## Abstract

*Background*. The use of statins is essential for aggressive lipid-lowering treatment in acute coronary syndrome (ACS) patients with dyslipidemia. Recently, elevation of sitosterol, a lipid absorption marker, was reported to be associated with premature atherosclerosis. The purpose of the present study was to examine the impact of ezetimibe, a selective intestinal cholesterol transporter inhibitor, in ACS patients. *Methods*. A total of 197 ACS patients were randomized to pitavastatin + ezetimibe (*n* = 100) or pitavastatin (*n* = 97). Low-density lipoprotein cholesterol (LDL-C) and sitosterol levels were evaluated on admission and after 12 weeks. *Results*. After 12 weeks, the pitavastatin + ezetimibe group showed a significantly greater decrease of sitosterol (baseline versus after 12 weeks; 2.9 ± 2.5 versus 1.7 ± 1.0 ng/mL, *P* < 0.001) than the pitavastatin group (2.7 ± 1.5 versus 3.0 ± 1.4 ng/mL). The baseline sitosterol level was significantly higher in patients with achieved LDL-C levels ≥ 70 mg/dL than in patients with levels < 70 mg/dL (3.2 ± 2.5 versus 2.4 ± 1.3 ng/mL, *P* = 0.006). *Conclusions*. Ezetimibe plus statin therapy in ACS patients with dyslipidemia decreased LDL-C and sitosterol levels more than statin therapy solo. Sitosterol Elevation was a predictor of poor response to aggressive lipid-lowering treatment in ACS patients.

## 1. Introduction

Aggressive lipid-lowering treatment is crucial to secondary prevention for patients with acute coronary syndrome (ACS). The effectiveness of lipid-lowering therapy using 3-hydroxy-3-methylglutaryl-coenzyme A reductase inhibitors (statins), targeting cholesterol synthesis in the liver, in reducing the risk of coronary events in this population is firmly established [1, 2].

The homeostasis of circulating cholesterol levels is modulated primarily by cholesterol synthesis and absorption. It has been suggested that the downregulation of cholesterol synthesis by statin therapy is compensated for by a rise in intestinal cholesterol absorption [3].

Accordingly, the beneficial effect on low-density lipoprotein cholesterol (LDL-C) lowering and subsequent reduction of cardiovascular disease in response to statin inhibition of cholesterol synthesis would be attenuated in individuals who demonstrated a greater rebound increase in cholesterol absorption [4].

A previous study mentioned the possibility that lower cholesterol absorption was associated with reduced cardiovascular events among elderly patients [5]. Moreover, recent studies have clarified the molecular mechanisms underlying intestinal cholesterol absorption, which would be another potential therapeutic target for dyslipidemia. Ezetimibe is a selective intestinal cholesterol transporter inhibitor that selectively inhibits cholesterol absorption by blocking the Niemann-Pick C1-like 1 receptor. In patients with sitosterolemia, who might experience up to 50-fold elevations in circulating sitosterol concentrations and show premature atherosclerosis despite normal cholesterol levels, ezetimibe successfully produced significant reductions in sitosterol concentrations [6].

The effect of ezetimibe on plasma sitosterol concentrations in patients with ACS, who are at high risk for secondary cardiovascular events, is currently unknown. Recent reports claimed that the success rate of the recommended optional goal of LDL-C level less than 70 mg/dL for very-high-risk patients was only 30% [7–9]. The combination of ezetimibe and statin inhibits both cholesterol synthesis and intestinal cholesterol absorption, resulting in approximately 10–20% greater reduction of LDL-C compared to statin alone [10–12]. Accordingly, the purpose of the present study was to examine the impact of adding ezetimibe on sitosterol in statin-treated ACS patients.

## 2. Methods

This was an open-label, randomized, prospective study. A total of 197 consecutive ACS patients, whose LDL-C levels were greater than 100 mg/dL, were enrolled from January 2010 to March 2013. Patients were randomized to an aggressive lipid-lowering group (pitavastatin + ezetimibe group; pitavastatin + ezetimibe 10 mg/day, *n* = 100) or a conventional lipid-lowering group (pitavastatin group; pitavastatin only, *n* = 97). Follow-up rate was 100% and serum samples at 12 weeks were obtained from all participants. The starting dose of pitavastatin was 2 mg and then left to the discretion of attending physicians. During the study period, the use of nonstudy antidyslipidemic agents was prohibited.

The serum lipid profile was assessed at the time of enrollment and 12 weeks after randomization in terms of total cholesterol (TC), LDL-C, triglycerides (TG), and high-density lipoprotein cholesterol (HDL-C). Levels of sitosterol and campesterol, as markers of cholesterol absorption, and lathosterol, as a cholesterol synthesis intermediate, were also measured at the time of enrollment and 12 weeks after randomization. All laboratory analyses were exclusively performed at SRL Inc., an external laboratory (Hachioji, Tokyo, Japan). LDL-C concentrations were estimated using Friedewald's formula [13]. The concentrations of sitosterol, campesterol, and lathosterol were measured on gas chromatography [14]. The impacts of ezetimibe on the changes in lipid profiles, cholesterol absorption, and synthesis markers were evaluated.

The study protocol was approved by the institutional review board and ethics committee. All participating patients provided their written, informed consent. Patient enrollment was carried out according to the principles of the Declaration of Helsinki.

Pearson correlation coefficients were used to assess the correlations between cholesterol absorption markers and individual baseline characteristics. Baseline clinical and laboratory characteristics were compared between those with and without elevated cholesterol absorption markers using two-way repeated measures ANOVA, the chi-square test, or paired and nonpaired *t*-tests. A value of *P* < 0.05 was considered significant. Statistical analyses were performed with statistical software (SPSS system ver. 20.0).

## 3. Results

The baseline characteristics of the study population are presented in Table 1. There were no significant differences, including the major risk factors of cardiovascular disease, between the two groups, except for the percentage of ST-segment elevation myocardial infarction on admission. Pitavastatin dosages were similar in the two groups (pitavastatin + ezetimibe group versus pitavastatin group: 2.3 ± 1.3 versus 2.1 ± 1.1 mg, *P* = 0.24).

**Figure 1:**
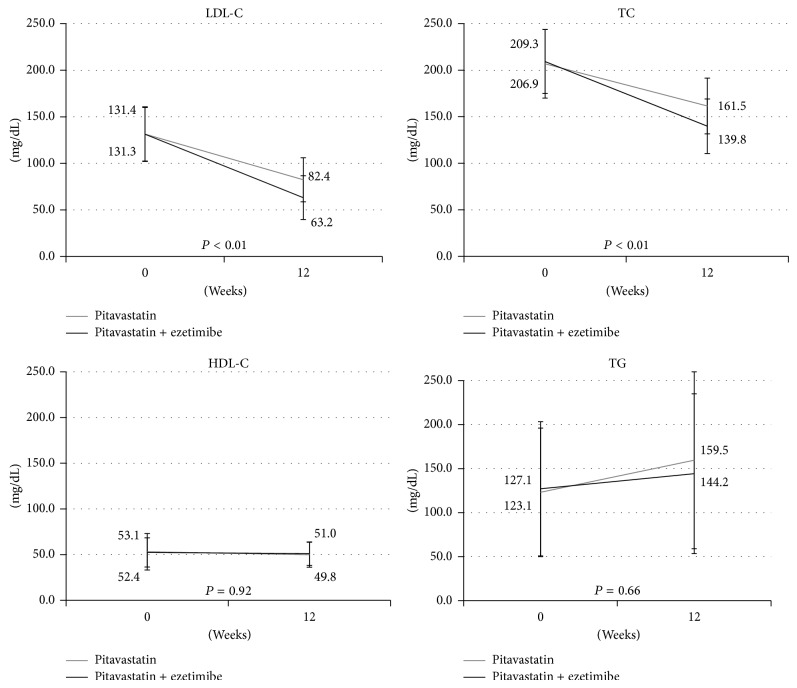
After 12 weeks of treatment, the serum levels of LDL-C and TC show a significant decrease in the pitavastatin + ezetimibe group compared to the pitavastatin group. In terms of the serum levels of HDL-C and TG, there are no significant changes in each group.

**Figure 2:**
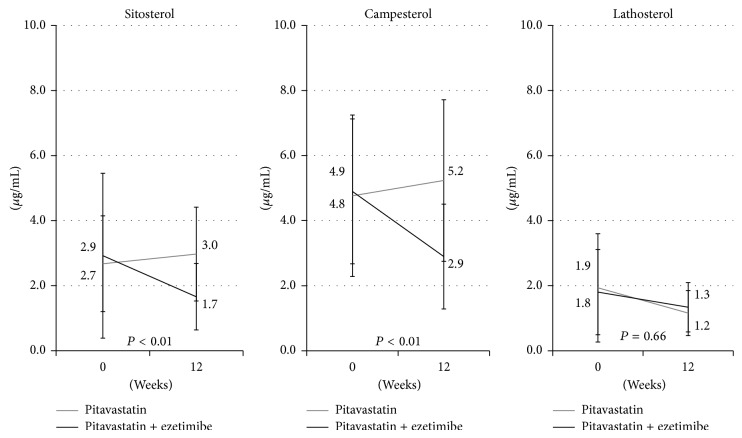
After 12 weeks of treatment, the serum levels of sitosterol and campesterol show a significant decrease in the pitavastatin + ezetimibe group compared to the pitavastatin group. The serum level of lathosterol decreases in both groups, but there is no significant difference between the two groups.

The serum levels of LDL-C, HDL-C, TG, TC, lathosterol, sitosterol, and campesterol are shown in Figures 1 and 2. The baseline lipid profiles were generally similar in the two groups. After 12 weeks of treatment, the serum levels of LDL-C, TC, sitosterol, and campesterol showed a significant decrease in the pitavastatin + ezetimibe group compared to the pitavastatin group.

The serum level of lathosterol decreased in both groups, with no significant difference between the groups. In terms of the serum levels of HDL-C and TG, there were no significant changes in each group.

**Figure 3:**
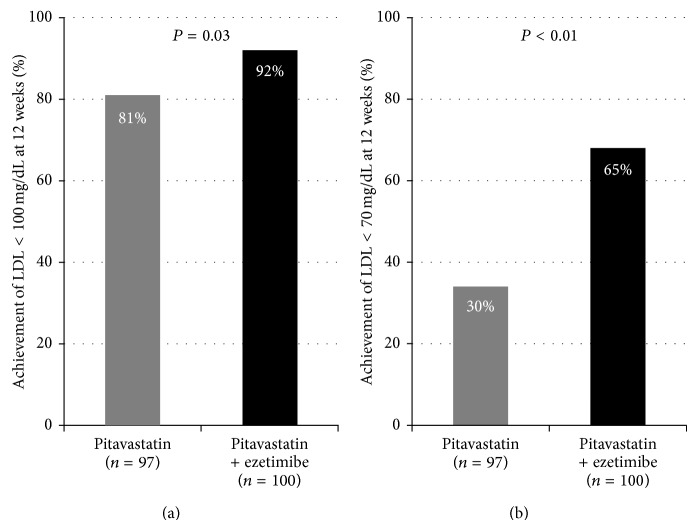
The achievement rate of LDL-C less than 100 mg/dL is the recommended target of the Japan Atherosclerosis Society guidelines, and the achievement rate of LDL-C less than 70 mg/dL is the recommended target of Adult Treatment Panel III (ATPIII) and European Atherosclerosis Society (EAS) guidelines. In each analysis, the achievement rate is significantly higher in the pitavastatin + ezetimibe group than in the pitavastatin group.

Figure 3 shows the achievement rate of LDL-C less than 100 mg/dL, which is the recommended target for high-risk patients in the Japan Atherosclerosis Society guidelines, moderate-risk patients in the Adult Treatment Panel III (ATPIII) and Japan Atherosclerosis Society guidelines, and the achievement rate of LDL-C less than 70 mg/dL, which is the recommended target of high-risk patients in ATPIII and European Atherosclerosis Society (EAS) guidelines. In each analysis, the achievement rate was significantly higher in the pitavastatin + ezetimibe group than in the pitavastatin group.

**Figure 4:**
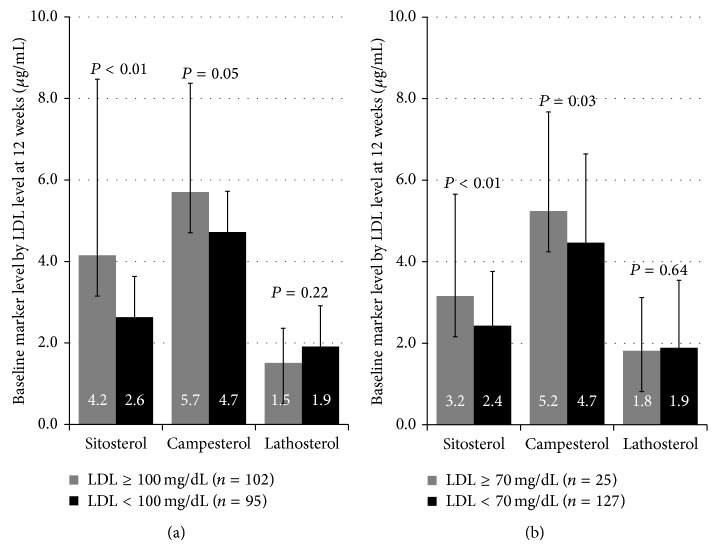
(a) The baseline levels of cholesterol synthesis and absorption markers according to the LDL-C levels at 12 weeks irrespective of treatment methods. The baseline levels of sitosterol and campesterol are significantly higher in patients whose LDL-C levels at 12 weeks are greater than or equal to 100 mg/dL than in patients whose LDL-C levels at 12 weeks are less than 100 mg/dL. In terms of the baseline level of lathosterol, there is no significant difference. (b) The results are the same when the cut-off of LDL-C is defined as less than 70 mg/dL at 12 weeks.

The baseline levels of cholesterol synthesis and absorption markers according to the LDL-C levels at 12 weeks were also examined irrespective of treatment methods. The baseline levels of sitosterol and campesterol were significantly higher in patients whose LDL-C levels at 12 weeks were greater than or equal to 100 mg/dL compared to patients whose LDL-C levels at 12 weeks were less than 100 mg/dL. In terms of the baseline level of lathosterol, there was no significant difference (Figure 4(a)). The results were the same when the cut-off of LDL-C was defined as less than 70 mg/dL at 12 weeks (Figure 4(b)).

**Figure 5:**
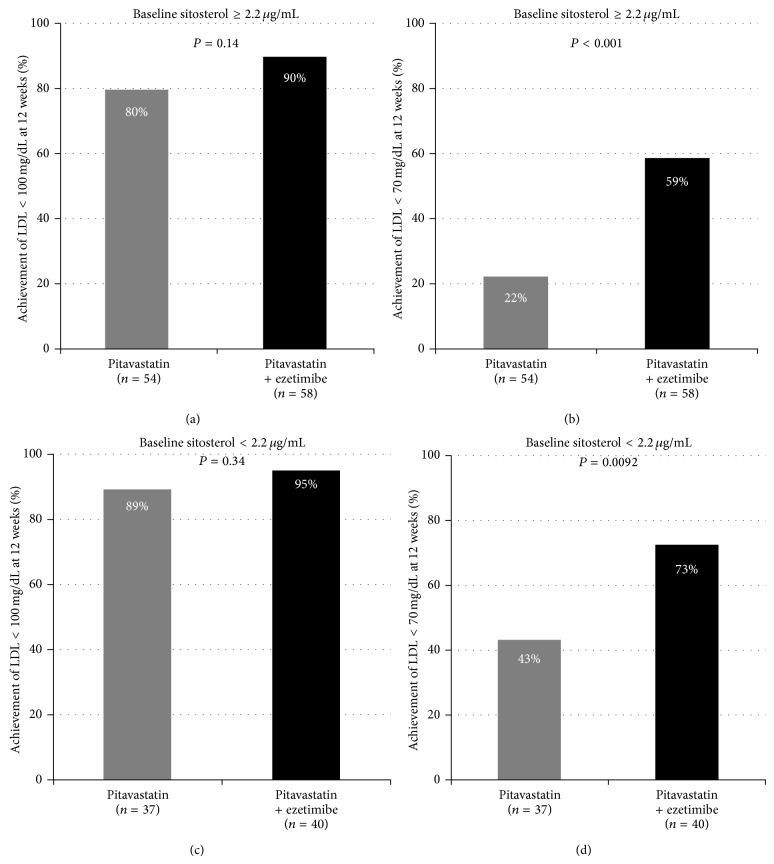
The impacts of baseline levels of sitosterol and lipid-lowering treatment strategy. (a) The achievement rate of LDL-C less than 100 mg/dL and (b) less than 70 mg/dL (b) in each treatment group whose baseline sitosterol is equal to or more than 2.2 *μ*g/mL. (c) The achievement rate of LDL-C less than 100 mg/dL and (d) less than 70 mg/dL in each treatment group whose baseline sitosterol is less than 2.2 *μ*g/mL.

Then, we performed additional analysis to examine the impact of baseline sitosterol concentration on lipid-lowering treatment (Figures 5(a)–5(d)). Sitosterol concentrations showed a left-skewed distribution, with a peak between 1.3 and 3.1 *μ*g/mL. Then, a sitosterol concentration of 2.2 *μ*g/dL was identified as the cut-off value and concentrations above this were defined as high. There were no significant differences between two treatment strategies in achievement of LDL-C less than 100 mg/dL (Figures 5(a) and 5(c)). However, pitavastatin + ezetimibe group showed higher achievement of LDL-C less than 70 mg/dL (Figures 5(b) and 5(d)). Moreover, in pitavastatin + ezetimibe group, there was no significant difference in achievement of LDL-C less than 70 mg/dL between high sitosterol group and low sitosterol group (59% versus 73%, *P* = 0.16), whereas there was a statistical difference in pitavastatin group (22% versus 43%, *P* = 0.033).

No clinical adverse events potentially related to statin and ezetimibe occurred during the study period. Laboratory data showed no clinically significant alterations of hepatic enzymes or creatine phosphokinase.

## 4. Discussions

The primary finding in the present study was a more significant reduction in serum levels of LDL-C, sitosterol, and campesterol after 12-week treatment with pitavastatin + ezetimibe in an ACS population, compared to monotherapy with pitavastatin. This study is short-term intervention trial to clarify the correlated fluctuations of the serum levels of sitosterol with the serum levels of LDL-C in ACS patients with dyslipidemia.

Coadministration of pitavastatin + ezetimibe resulted in a 15.3% reduction of the serum LDL-C level in patients with ACS. Because the dosage of pitavastatin was similar, the degree of restoration in LDL-C synthesis was considered to be identical. Thus, inhibiting LDL-C absorption by ezetimibe would be the main reason for the decreased serum LDL-C level.

Statins are the mainstay of therapy for dyslipidemia. In patients with stable coronary heart disease, the Treating to New Target (TNT) study showed that intensive lipid-lowering therapy with 80 mg/dL of atorvastatin per day, targeting LDL-C less than 75 mg/dL, provided a 22% relative risk reduction compared with standard lipid-lowering therapy with 10 mg/dL of atorvastatin per day, targeting LDL-C less than 100 mg/dL [15]. However, the Lipid Treatment Assessment Project 2 showed that the success rate in reaching the more aggressive optional goal of LDL-C less than 70 mg/dL among patients who were at very high risk of cardiovascular events was only 30% in 2007 [16]. In the present study, the achievement rate of LDL-C less than 70 mg/dL, recommended in ATPIII and EAS guidelines, was 65% in the pitavastatin + ezetimibe group, which was 30% more than that in the monotherapy with pitavastatin group. Accordingly, the addition of ezetimibe to statin is considered to be a good option for aggressive lipid-lowering treatment.

Although the potential significance of plant sterol elevations in the general population is still less clear, there are several reports that a high plant sterol concentration is atherogenic and associated with coronary artery disease [17–20]. A more recent study showed that the campesterol to lathosterol ratio may be related to plaque vulnerability in the coronary artery with significant stenosis [21].

Previous studies have already suggested the importance of inhibiting intestinal cholesterol absorption for the primary and secondary prevention of cardiovascular events. In a subanalysis of the 4S study, the prevention of clinical events by statins was adequate in patients with lower cholesterol absorbers, whereas it was suboptimal in patients with higher cholesterol absorbers [22]. The results from a nested case-control analysis of the Prospective Cardiovascular Munster (PROCAM) study and the prospective study of pravastatin in the elderly at risk (PROSPER) showed that plasma sitosterol elevations are associated with an increased risk of coronary events in patients with coronary heart disease [4, 23].

It is important to clarify the clinical characteristics of patients who would likely benefit from their respective therapies. In hyperlipidemic patients with high level of baseline cholesterol absorption markers, previous study reported that ezetimibe added to statin therapy was the most effective strategy for LDL-C lowering [24]. However, no previous studies evaluated the relationship between cholesterol absorption and synthesis among ACS patients, who are thought to have greatest potential benefit from aggressive lipid-lowering treatment, targeting LDL-C less than 70 mg/dL. The present study revealed, for the first time in ACS patients, that the baseline levels of cholesterol absorption markers were significantly higher in patients whose LDL-C levels at 12 weeks were greater than or equal to 70 mg/dL compared to patients whose LDL-C levels at 12 weeks were less than 70 mg/dL. Cholesterol absorption enhancement seemed to have a negative effect on the current aggressive lipid-lowering strategy. As the rationale for the above, ACS patients who show enhanced cholesterol absorption are considered reasonable targets of ezetimibe plus statin treatment.

There were some limitations in the present study. First, the number of patients in the present study was small. Second, the observation period was short. To prove the benefit in clinical outcomes, a larger study population and longer follow-up are needed.

In conclusion, ezetimibe produced significant and progressive reductions in LDL-C and cholesterol absorption markers in ACS patients with dyslipidemia. The baseline levels of cholesterol absorption markers would be useful for the selection of ACS patients who will have resistance to conventional lipid-lowering treatment.

## Figures and Tables

**Table 1:** Baseline characteristics of patients in the study groups.

Variable	Pitavastatin + ezetimibe	Pitavastatin	*P* value
	(*n* = 100)	(*n* = 97)	
Age (y)	65.2 ± 11.3	66.6 ± 12.0	0.4
Men	70 (70.0%)	75 (77.3%)	0.24
BMI (kg/m^2^)	23.8 ± 3.3	23.7 ± 4.8	0.86
Hypertension	74 (74.0%)	67 (69.1%)	0.44
Diabetes mellitus	33 (33.0%)	31 (32.0%)	0.88
Smoker	55 (55.0%)	57 (58.8%)	0.59
Family history of CAD	26 (26.0%)	24 (24.7%)	0.84
Prior angina or myocardial infarction	26 (26.0%)	25 (25.8%)	0.97
Prior PCI	22 (22.0%)	24 (24.7%)	0.65
Prior CABG	5 (5.0%)	9 (9.3%)	0.24
Diagnosis on admission			
STEMI	48 (48.0%)	32 (33.0%)	0.03
NSTEMI	5 (5.0%)	11 (11.3%)	0.1
UAP	47 (47.0%)	54 (55.7%)	0.22
TIMI risk score	3.6 ± 1.5	3.9 ± 1.7	0.19
Medication			
Statin	23 (23.0%)	26 (26.8%)	0.54
Antiplatelet agent	38 (38.0%)	49 (50.5%)	0.08
ACEI/ARB	48 (48.0%)	38 (39.2%)	0.21
CCB	36 (36.0%)	35 (36.1%)	0.99
*β*-blocker	15 (15.0%)	27 (27.8%)	0.03
Baseline laboratory data			
LDL-C (mg/dL)	130.1 ± 31.3	131.9 ± 27.7	0.67
HDL-C (mg/dL)	45.6 ± 12.0	46.2 ± 11.0	0.72
Triglycerides (mg/dL)	122.9 ± 71.3	124.4 ± 71.7	0.88
eGFR (mL/min/1.73 m^2^)	57.2 ± 16.0	58.9 ± 18.3	0.49
UA (mg/dL)	5.8 ± 1.5	5.9 ± 1.4	0.63
HbA1c (%)	6.2 ± 1.5	6.3 ± 1.3	0.62

BMI: body mass index, CAD: cardiovascular disease, PCI: percutaneous coronary intervention, CABG: coronary artery bypass grafting, STEMI: ST-segment elevation myocardial infarction, NSTEMI: non-ST-segment elevation myocardial infarction, UAP: unstable angina pectoris, TIMI risk score: thrombolysis in myocardial ischemia risk score, ACEI: angiotensin-converting enzyme inhibitor, ARB: angiotensin receptor blocker, CCB: calcium channel blocker, LDL-C: low-density lipoprotein cholesterol, HDL-C: high-density lipoprotein cholesterol, eGFR: estimated glomerular filtration rate, UA: uric acid, and HbA1c: hemoglobin A1c.
